# Hazard Assessment of Debris-Flow along the Baicha River in Heshigten Banner, Inner Mongolia, China

**DOI:** 10.3390/ijerph14010030

**Published:** 2016-12-29

**Authors:** Chen Cao, Peihua Xu, Jianping Chen, Lianjing Zheng, Cencen Niu

**Affiliations:** 1 College of Construction Engineering, Jilin University, Changchun 130026, China; caochen14@mails.jlu.edu.cn (C.C.); xuph@jlu.edu.cn (P.X.); niucencen@jlu.edu.cn (C.N.); 2 Construction Engineering College, Changchun Sci-Tech University, Changchun 130600, China; zhengljcc@gmail.com

**Keywords:** 3S technologies, cloud model, analytical hierarchy process, entropy method

## Abstract

This study focused on a cloud model approach for considering debris-flow hazard assessment, in which the cloud model provided a model for transforming the qualitative and quantitative expressions. Additionally, the entropy method and analytical hierarchy process were united for calculating the parameters weights. The weighting method avoids the disadvantages inherent in using subjective or objective methods alone. Based on the cloud model and component weighting method, a model was established for the analysis of debris-flow hazard assessment. There are 29 debris-flow catchments around the pumped storage power station in the study area located near Zhirui (Inner Mongolia, China). Field survey data and 3S technologies were used for data collection. The results of the cloud model calculation process showed that of the 29 catchments, 25 had low debris-flow hazard assessment, three had moderate hazard assessment, and one had high hazard assessment. The widely used extenics method and field geological surveys were used to validate the proposed approach. This approach shows high potential as a useful tool for debris-flow hazard assessment analysis. Compared with other prediction methods, it avoids the randomness and fuzziness in uncertainty problems, and its prediction results are considered reasonable.

## 1. Introduction

Debris-flow (DF) is a common nature disaster that always occurs in the catchments of mountain areas. The high mobility of DF has a great impact on people’s lives and properties, as well as infrastructure [1,2,3,4]. Since the 1970s, the annual deaths caused by DF in China have reached more than 3700 [5]. The direct economic losses are estimated at about 3.3 billion to 3.6 billion CNY per year [6,7,8].

Researchers have thus paid close attention to the topic of DF hazard assessment. Eldeen [9] considered that the DF was similar to floods. He introduced the flood hazard assessment method into DF hazard assessment. Based on a scoring method and the parameter superposition principle, Hollingsworth and Kovacs [10] mainly considered three parameters, including lithology, slope and ravine density. Nowadays, many DF hazard assessment methods are based on the parameter superposition principle. Different kinds of nonlinear mathematics methods were introduced to assess the DF hazards and hazard assessment [5,11,12]. Liu and Tang [13] analyzed the influencing parameters that decide the DF hazard degree with principal ingredient analysis. In quantitative approaches, Carrara [14] adopted statistical analysis to solve the problem of subjectivity. Zhang et al. [4,8] used extenics and fuzzy C-means algorithm to determine the Wudongde dam region debris-flow catchments hazard degrees by analyzing the influencing parameters. Liang et al. [15] used a Bayesian Network to assess DF hazard degrees on a national scale. Niu et al. [5] employed a stepwise discriminant analysis to reduce 17 parameters to five major parameters, simplifying the calculation of DF hazard assessment.

Since the end of the 20th century, 3S technologies has been introduced to DF hazard assessment [15,16]. 3S technologies refers to data collectively provided by geographic information system (GIS), global positioning system (GPS), and remote sensing (RS). It is a powerful technology for spatial analysis and data acquisition. For large-scale debris flow catchments, 3S technologies are useful for studying areas that humans cannot reach. Han [17] proposed a computer numerical technology to replace the traditional physical simulation for DF quantitative hazard assessment. In recent years, researchers have combined 3S technologies and nonlinear methods to perform DF hazard assessments [11,15,18,19,20]. The introduction of 3S technologies in DF hazard evaluation has made the obtaining of the spatial data more and more efficient and accurate.

This study focused on a hazard assessment of a series of debris-flow catchments along the Baicha River near the town of Zhirui in Heshigten Banner, Inner Mongolia, China. Field surveys are necessary and generally reliable, although artificial errors in field survey data might sometimes lead to inaccuracies that result in values near a threshold being classified into the incorrect category. Fuzziness in a classification process is not always easy to address and it is a feature that requires further attention. In conventional fuzzy-based approaches, the sectional fuzzy function, including its types, boundaries, and parameters, should be determined separately for each evaluation parameter. DFs represent complex nonlinear geological disasters, the hazard assessment of which is affected by many parameters. It makes it difficult to distinguish boundaries qualitatively. This paper presents an innovative method based on a cloud model (CM), which has the ability to express fuzziness and randomness to address this shortcoming. The distance function was applied to unite the entropy method and analytical hierarchy process (EAHP) to obtain the weights of the parameters that influence DF hazard. The EAHP method avoids the disadvantages inherent in using subjective or objective methods in isolation. There have been few previous studies on DF hazard assessment using a CM-based approach. This paper introduces an innovative CM-based approach for the assessment of the DF hazard of 29 catchments in Inner Mongolia, China. The approach was validated using the extenics method and field surveys.

## 2. Study Area

**Figure 1:**
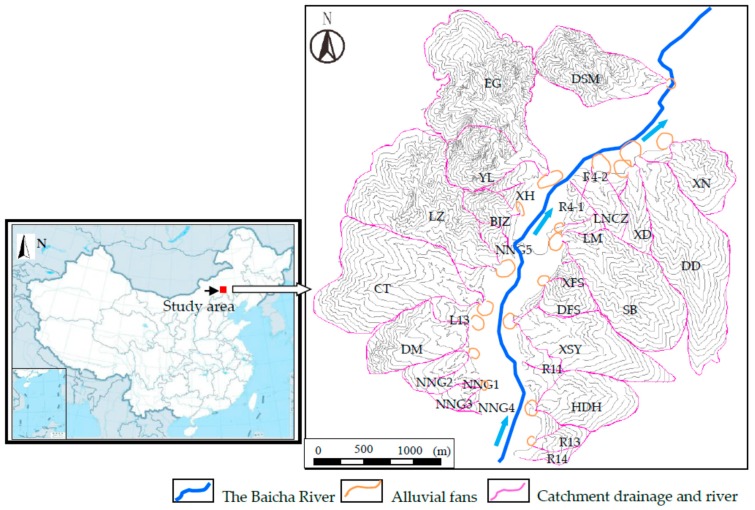
Geographical position of the investigated 29 debris-flow catchments.

The study area of the pumped storage power station is located 3 km north of the town of Zhirui in Heshigten Banner, Inner Mongolia, China. The Baicha River flows from the SSW to the NNE through the lower reservoir before reaching Gaojiayingzi town. As shown in Figure 1, there are 29 DF catchments distributed along both banks of the Baicha River. Loose material is prone to discharge from the DF catchments, accumulating on the banks or cascading into the Baicha River itself. Therefore, it was considered necessary to conduct a hazard assessment of the DFs.

**Figure 2:**
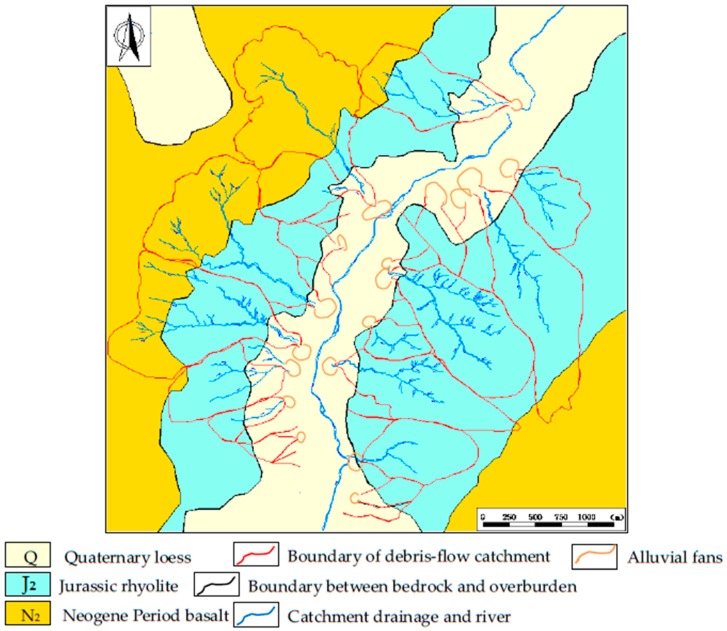
Geological map of the study area.

**Figure 3:**
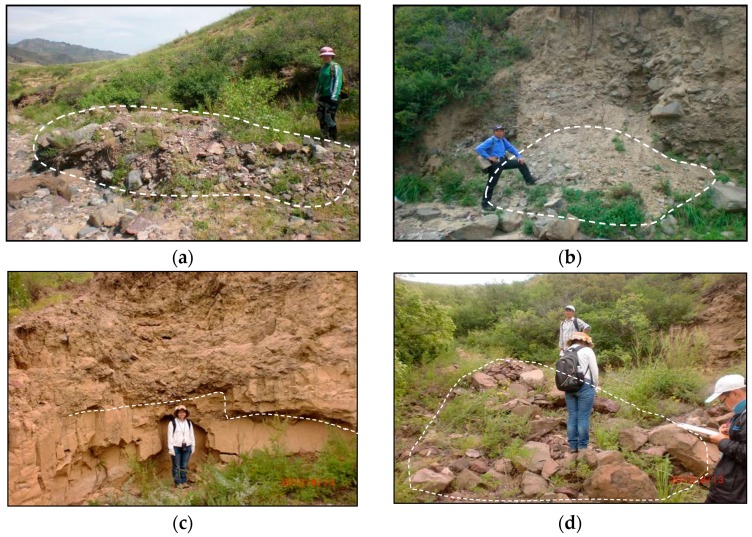
Field survey: (**a**) loose deposits in EG catchment; (**b**) collapsed mass in EG catchment; (**c**) lateral erosion in XN catchment and (**d**) loose gravel in XN catchment.

The elevation of this area ranges from 1100 to 1700 m above sea level. It lies within a middle-mountain district and the trend of the mountains is NE. Neogene basalts covering the surface of the mountains has led to the tops of the mountains to be shaped as flat terraces. The slope angles of the mountains range from 30° to 50°. The Baicha River is U-shaped and the width of the bed ranges from 200 to 500 m above sea level. There are three main types of exposed strata including Quaternary (Q) loess, Neogene (N_2_) basalt, and Jurassic (J_2_) rhyolite. The loess, basalt, and rhyolite are distributed around the Baicha River, as shown in Figure 2. No regional faulting is found within the study area. We used the global positioning system (GPS) to locate the field phenomenon, such as loose deposits, collapsed mass, and loose gravel distributed within the 29 DF catchments. Lateral erosion occurs via hydrodynamic scouring (Figure 3).

The study area is characterized by a temperate continental monsoon climate. The southern part is a warm–cool semiarid agroclimatic zone. The temperature in January is −24–−19 °C, and in July it is 17–21 °C. The northern part is a warm–cold wet semi forested climatic zone. The average annual temperature is 2–5 °C. The minimum and maximum temperatures are −18 and 21 °C, respectively. Annual rainfall in Heshigten Banner is 250–540 mm. In the forested areas of Dajuzi District, the annual average rainfall can reach 531.8 mm (maximum rainfall was 741.6 mm in 1959). In the pastoral areas, the annual average rainfall is 350–400 mm. The annual rainfall in the agricultural areas increases from east to west with most districts receiving 300–390 mm.

## 3. Methodology

### 3.1. Cloud Model

Li [21] proposed a cloud model (CM), which is a conversion model between qualitative and quantitative numerical data. Based on probability theory and fuzzy mathematics, the CM explores the relationship between fuzziness and randomness. The CM is defined as follows. It is supposed that *A* is a quantitative domain expressed by accurate numbers and *C* is a quality concept in *A*. There exists a matching certainty degree *μ*(*x*) ∈ [0, 1] to *C* for arbitrary *x*∈*A.* The distribution of *x* in *A* is called a cloud and each *x* is called a cloud drop:

(1) μ: A→[0, 1], ∀ x∈A, x→μ(x)

A CM can be characterized by three quantitative characteristics [21,22]: expectation (*E_x_*), entropy (*E_n_*), and hyper-entropy (*H_e_*). *E_x_* is the mathematical expectation that a cloud drop belongs to a concept in the universal. It can be regarded as the most representative and typical sample of the qualitative concept. *E_n_* represents the uncertainty measurement of a qualitative concept, the larger its value, the greater the fuzziness and randomness of the concept. It not only reflects the dispersing extent of cloud drops, but also the measurement of fuzziness. *H_e_* is a measure of the randomness and fuzziness of *E_n_*, which reflects the uncertainty aggregation degree of the cloud drop. Its value indirectly reflects the thickness of the cloud and is determined by randomness and fuzziness of entropy. The larger the value of *H_e_*, the larger the randomness of the membership degree and the thickness of the cloud drops [23].

**Figure 4:**
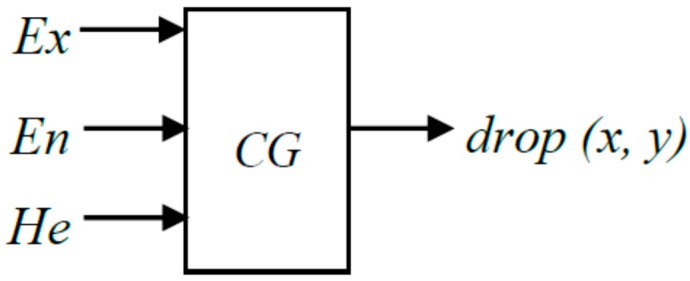
Forward cloud generator (CG).

A cloud generator establishes a relationship between the qualitative and the quantitative characteristics. The cloud generator mainly includes a forward cloud generator and a backward cloud generator. The forward cloud generator is a basic cloud generator algorithm, which realizes the mapping of qualitative to quantitative characteristics. The input is the quantitative characteristics of the cloud (*E_x_*, *E_n_*, *H_e_*) and cloud drops *N.* The output is the quantitative locations of the *N* cloud drops in the cloud and the certainty degree represented by each cloud drop, as shown in Figure 4.

The specific algorithm is as follows:

1. Generate a normally distributed random number *y_i_*, whose mean value is *E_n_* and standard deviation is *H_e_*.
2. Generate a normally distributed random number *x_i_*, whose mean value is *E_x_* and standard deviation is *y_i_*.
3. Let *x_i_* be a specific quantitative value of qualitative characteristics, named a cloud drop.
4. Calculate the certainty degree of the random number: $\mu =e^{\frac{-{\left(x_{i}-E_{x}\right)}^{2}}{2y_{i}{}^{2}}}$.
5. Output a cloud drop (*x_i_*, *μ_i_*).
6. Repeat steps 1–5 until N cloud drops are generated and all the cloud drops comprise the cloud and realize a qualitative presentation.

### 3.2. Weighting Methods

(1) The analytical hierarchy process (AHP) is a multi-objective decision analysis method proposed by Saaty [24]. The weights of these criteria are defined after they are ranked according to their relative importance. Thus, once all the criteria are sorted in a hierarchical manner, a pairwise comparison matrix for each criterion is created to enable a significance comparison. The relative significances of the criteria are evaluated on a scale of 1–9, indicating less importance to greater importance. The steps of the AHP for weighting are as follows:

1. The first step is to build the hierarchical structure of the target problem.
2. Saaty [24] proposed a scaling method to score the parameters in each layer. By comparing the importance of the parameters in each level, *a_ij_* is used to present the ratio of *x_i_* and *x_j_*, which builds the judgment matrix *A* = (*a_ij_*):
  (2) $$A={\left(a_{ij}\right)}_{n\times n}=\left[\begin{array}{cc} \begin{array}{cc} a_{11} & a_{12}\\ a_{21} & a_{22} \end{array} & \begin{array}{cc} \cdots & a_{1n}\\ \cdots & a_{2n} \end{array}\\ \begin{array}{cc} \cdots & \cdots \\ a_{n1} & a_{n2} \end{array} & \begin{array}{cc} \cdots & \cdots \\ \cdots & a_{nn} \end{array} \end{array}\right]$$
3. The judgment matrix needs to satisfy the following equation: 𝐴𝜔 = 𝜆_max_𝜔, where *ω* is the maximum characteristic vector matching the feature vector of judgment matrix *A*. The weight value can be obtained after normalizing the feature vector.
4. In order to analyze whether the weighting distribution is reasonable, a consistency check is needed. The consistency parameter (*CI*) can be obtained using the following equation:
  (3) $$CI=\frac{\lambda_{\max}-n}{n-1}$$

The random consistency ratio can be obtained using the following equation:

(4) CR=CI/RI

where *n* is the order of the random index (*RI*). The value of *RI* can be obtained from Zhang [4]. If *CR* is lower than the threshold (0.1), the weighting consistency is affirmed, which means the weighting of the parameters is reasonable.

(2) The entropy method [25] is an objective weighting method that determines the weights of the parameters based on measured values. In information theory, entropy is a measure of the uncertainty of the formulation in terms of probability theory; greater amounts of information mean smaller degrees of uncertainty and entropy, and vice versa. According to the characteristics of entropy, the randomness and degree of disorder can be represented by the value of entropy. The discrete degree of a parameter is represented by its entropy value. The greater the discrete degree is, the larger the influence of the parameter on the comprehensive evaluation. If it is assumed there are *m* proposals to be assessed and *n* evaluation parameters, an original data matrix *X* = (*x_ij_*)*_m_*_×*n*_ can be formed. For parameter *x_j_*, the greater the parameter value *X_ij_*, the more important the role that parameter plays in the comprehensive evaluation. If the values of an evaluation parameter are all equal, then that parameter does not play a role in the comprehensive evaluation. The occurrence of a DF is influenced by many parameters. It is difficult to describe DF hazard assessment using a traditional objective weighting method. However, the entropy theory can be used to assess the DF hazard. In this way, the disorder degree of the DF is expressed and given objective weights based on objective measured data. The steps of the weighting calculation using the entropy method are as follows:

1. Standardize the data. After standardizing the original data matrix *X* = (*x_ij_*)*_m_*_×*n*_, obtain the normalized matrix *R* = (*r_ij_*)*_m_*_×*n*_. The normalizing equations are:
  The greater the better:
  (5) $$r_{ij}=\frac{X_{ij}-\min\{X_{ij}\}}{\max\left\{X_{ij}\right\}-\min\left\{X_{ij}\right\}},\left(i=1,2,\ldots ,n;j=1,2,\ldots ,m\right)$$
  The smaller the better:
  (6) $$r_{ij}=\frac{\max\{X_{ij}\}-X_{ij}}{\max\left\{X_{ij}\right\}-\min\left\{X_{ij}\right\}},\left(i=1,2,\ldots ,n;j=1,2,\ldots ,m\right)$$
2. Calculate the *i*-th catchment *j*-th factor proportion:
  (7) $$P_{ij}=\frac{X_{ij}}{\overset{n}{\underset{i=1}{\sum }}X_{ij}}\left(j=1,2,\ldots ,m\right)$$
3. Calculate the information entropy of the factor:
  (8) $$e_{j}=-k\overset{n}{\underset{i=1}{\sum }}Y_{ij}\times \ln Y_{ij}$$
  where *k* > 0, ln is the natural logarithm, *e_j_* ≥ 0, *k* is relative to the DF catchment number *m*, *k =* 1/ln*m*, and 0 ≤ *e* ≤ 1.
4. Calculate the information entropy redundancy:
  (9) $$g_{j}=1-e_{j}$$
  As for the *j*-th factor, the greater the difference of the factor value *X_ij_*, the greater the role that factor plays and the smaller the entropy value *e_j_*.
5. Calculate the factor weights:
  (10) $$W_{j}=\frac{g_{j}}{\overset{m}{\underset{j=1}{\sum }}g_{j}},\left(i=1,2,\ldots ,m\right)$$

(3) The determination of the AHP judgment matrix is based on expert knowledge and the weighting of the entropy method depends on objective data. The objective and subjective weighting methods are combined into the EAHP weighting method using the distance function.

If it is assumed the weighting acquired by AHP is $\omega_{i}^{,}$ and the weighting calculated by the entropy method is $\omega_{i}^{,,}$, then the distance function of the two methods is *d* ($\omega_{i}^{,}$, $\omega_{i}^{,,}$), whose equation is as follows:

(11) $$d\left(\omega_{i}^{,},\omega_{i}^{,,}\right)={\left[\frac{1}{2}\overset{n}{\underset{i=1}{\sum }}{\left(\omega_{i}^{,}-\omega_{i}^{,,}\right)}^{2}\right]}^{\frac{1}{2}}$$

Assume the combined weighting is $\omega_{z}$, which is a linear weighting of $\omega_{i}^{,}$ and $\omega_{i}^{,,}$:

(12) $$\omega_{z}=\alpha \omega_{i}^{,}+\beta \omega_{i}^{,,}$$

where *α* and *β* are two types of weighting partition coefficient.

In order to get the partition coefficients, the distance function is used, the equations for which are:

(13) $$d\left(\omega_{i}^{,},\omega_{i}^{,,}\right)={\left(\alpha -\beta \right)}^{2}$$

(14) α+β=1

The partition coefficients are obtained using Equations (13) and (14) and then substituted into Equation (12) to obtain the comprehensive weighting.

## 4. Debris-Flow Hazard Assessment

### 4.1. Evaluation Parameters

Previous research of DF hazard assessment has largely considered influencing parameters in terms of only four aspects: topography, meteorological and hydrological setting, geological setting, and anthropogenic activity. However, many other parameters influence DFs; therefore, it is necessary to include an appropriate selection for an accurate hazard assessment. Based on the principles of parameter selection for DF hazard assessment and other relevant studies [4,8,15,16,26,27,28,29,30,31,32,33,34,35,36,37,38], this study selected 11 influencing parameters based on the characteristics of the study area, as described below:

#### 1. Catchment area (*x*_1_/km^2^)

The catchment area reflects the confluence of the DF catchment and it represents the potential scale of the DF. However, it does not mean that larger catchment areas have greater hazard to DFs. Catchments of more than 100 km^2^ appear susceptible to flooding. Here, catchment area was selected as an appropriate parameter, and the threshold was set as 0–50 km^2^. Given that most of the 29 DF catchments within the study area are <0.5 km^2^, the low hazard range was set as 0.0–0.5 km^2^.

#### 2. Main channel length (*x*_2_/km)

The main channel length is the total length of the DF source and transportation areas. Long main channel lengths lead to larger-scale DFs with greater destructive power. Chang [16,20,39] regarded this as a very important parameter in terms of degree of risk, vulnerability to DFs, and hazard assessment. The scheme of Liu [40] was used for the classification of the degree of this parameter.

#### 3. Maximum elevation difference (*x*_3_/km)

This represents the difference between the maximum and minimum elevations of the catchment above sea level. It reflects the ability of the DF to carry solid material and provides an assessment of the catchment’s potential energy. In China, mountains are divided into four types based on elevation difference: <0.2 km are hills, 0.2–0.5 km are middle–low mountains, 0.5–1.0 km are high mountains, and >1.0 km are extremely high mountains.

#### 4. Ravine density (*x*_4_/km·km^−2^)

Ravine density is the ratio of the total ravine length to the total catchment area. The ravine reflects the geological structure, lithology, and the degree of rock weathering comprehensively because ravines always develop in weak areas [8]. The range of lateral erosions and retrogressive are reflected by ravine. With the development of the debris flows, the ravine density would gradually increase. Generally, higher ravine density corresponds to higher cumulative debris volume.

#### 5. Curvature of the main channel (*x*_5_)

According to previous studies [41,42,43], *x*_5_ reflects the discharge situation of a DF. It represents the ratio of the curve length of the main channel to a straight length joining the same two measurement points. Erosion by water has an impact on the stability of slopes. If slope failure happens, the supply of loose material to the channel will be increased [44]. Here, the classification method of Liu [40] was used for this parameter.

#### 6. Loose material length supply ratio (*x*_6_)

This factor is defined as the ratio of loose material length along a channel to total channel length. It reflects the supply range and volume of loose material comprehensively. A higher *x*_6_ value results in a wider range of loose the materials, which could make more debris volume added in the debris flow. Thus, the higher the *x*_6_ value is, the higher the threat of debris flow. It directly influences the DF scale, which has a close relationship with DF hazard assessment. The number of possible slope phenomena and potential volumes of mobilized material from source areas to the main river [37]. Previous work suggests that a supply ratio of <0.1 indicates a catchment with no DF hazard, 0.1–0.3 is slightly hazardous, 0.3–0.6 is moderately hazardous, and >0.6 is highly hazardous [40].

#### 7. Twenty-four-hour maximum rainfall (*x*_7_/mm)

Water is a vital component of a DF because it is the transport medium. In the study area, rainfall is the only source of water and it is one of the main parameters that induce DFs [39,45]. Debris flows are mainly triggered by heavy rains [38]. The assessment of debris flow relies mainly on rain intensity [29].

#### 8. Population density (*x*_8_/per·km^−2^)

Anthropogenic activities have a close relationship with DFs and many DFs have been triggered by the negative consequences of socioeconomic development. Human activity, such as deforestation and slope cutting, accelerates the formation and development of debris flows seriously [8]. Landslides and slag caused by constructing roads and mining will increase the amount of loose material, thus increases the hazardous of debris flow. This parameter represents the strength of anthropogenic activities.

#### 9. Loose material volume (*x*_9_/10^4^ m^3^)

The abundance of loose material is a necessary condition that determines the scale of DFs [5,36]. Catchments with steep mountains experience large sediment pulses from hillslopes which are stored in headwater channels [35]. Within the catchments, this parameter was assessed using measurement and a laser rangefinder.

#### 10. Outbreak frequency (*x*_10_/%)

Even on a small scale, if the DF outbreak frequency is high, it could still cause considerable harm to the population and severe damage to properties within the catchment. Outbreak frequency represents the occurrence of DFs per 100 years. Considering the actual conditions of China, this study set the highest degree as once per year and the lowest degree as once every 10 years.

#### 11. Main channel gradient (*x*_11_)

Previous studies have shown that the average gradient of the main channel has a close relationship with the triggering of DFs [39,41,45,46,47,48,49]. Higher gradients imply greater DF velocity and greater energy for destruction. The main channel gradients in this study were obtained using field survey data and geographic information system technology. The classification scheme was largely based on Zhang [4].

**Figure 5:**
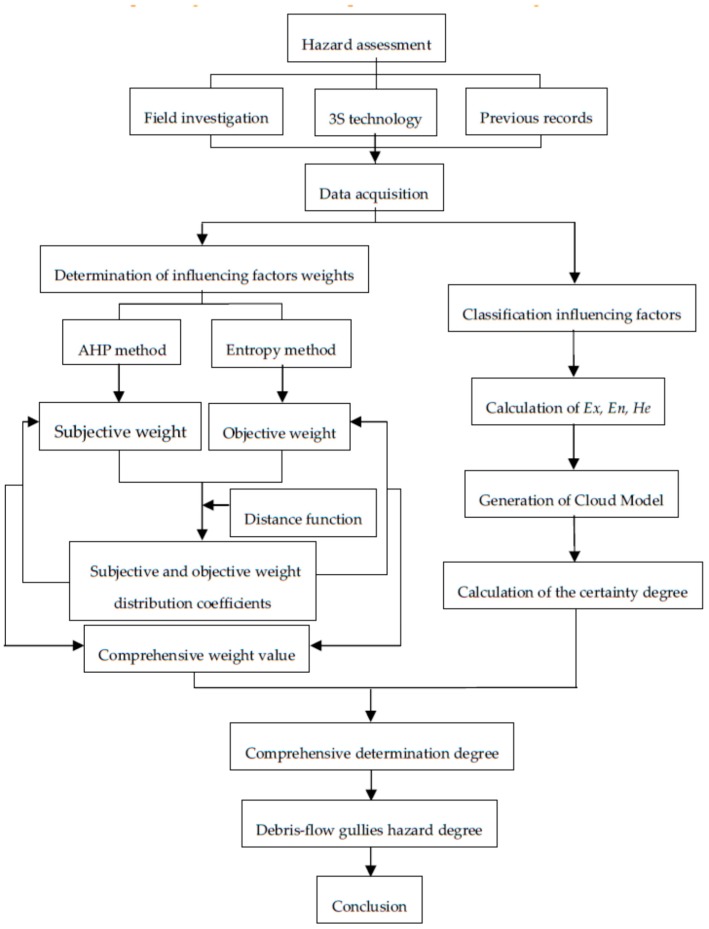
Debris-flow hazard assessment process flow chart.

The corresponding flow chart of the process is shown in Figure 5.

### 4.2. Establishment of the Cloud Model

Based on the above, a CM was used to establish a DF hazard assessment model. Following CM theory, the quantitative characteristics (*E_x_, E_n_, H_e_*) were calculated using the following equations:

(15) $$E_{x}^{p}=\frac{M_{p}+N_{p}}{2}\;p\left(p=\;1,2,\ldots ,P\right)$$

(16) $$E_{n}^{p}=\{\begin{array}{c} \frac{E_{x}^{2}-E_{x}^{1}}{3}p=1\\ \frac{E_{x}^{p}-E_{x}^{p-1}}{3}p\geq 2 \end{array}\;p\left(p=\;1,2,\ldots ,P\right)$$

(17) $$H_{e}=\beta$$

where *p* is the hazard degree of the DF catchment, *M_p_* and *N_p_* are the upper boundary and lower boundary of a certain evaluation parameter of the *i*-th degree, and *β* is a constant. The value of *H_e_* indirectly reflects the thickness of the cloud. In order to obtain accuracy certainties, *β* was set as 0.01 in this study. The boundaries are shown in Table 1.

**Figure 6:**
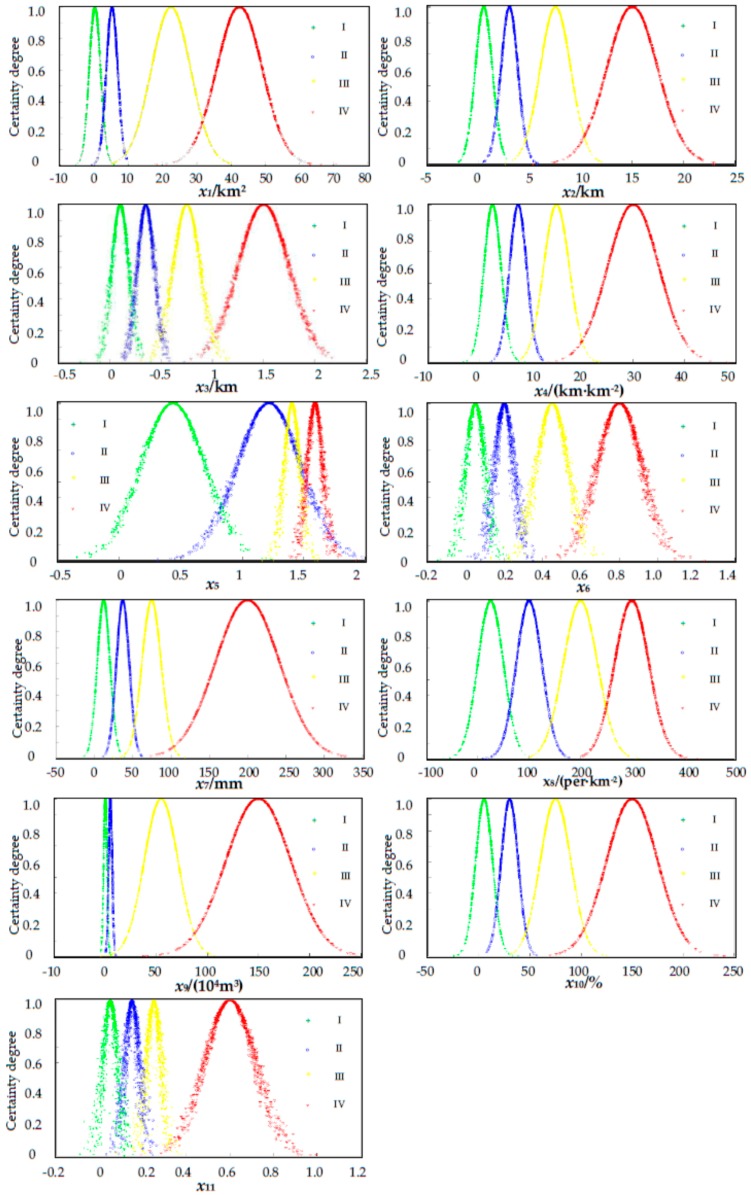
Debris-flow hazard assessment influencing parameters cloud model evaluation charts. I: low; II: moderate; III: high and IV: extremely high.

The forward cloud generator was applied to generate clouds with the quantitative characteristics. The clouds, which are represented as low (I), moderate (II), high (III), and extremely high (IV), are shown in Figure 6. The CM has the ability for qualitative and quantitative transformation to determine the hazard degree for each parameter. Furthermore, the CM-based approach also considers the fuzziness and randomness of each parameter in adjacent degrees. Table 2 is the basic data that obtained through field investigation and 3S technologies. The three-dimensional (3-D) characteristics of the 29 DF catchments were obtained through a digital elevation model (DEM), which is constructed to obtain factors related to elevation. Meanwhile, the catchment area is also obtained through GIS software.

### 4.3. Calculation of Weights Using the EAHP Method

Considering that different evaluation parameters have different contributions to DF hazard, it is important to develop an appropriate weighting method for the assessment. To begin, the AHP weighting method can be used to calculate subjective weights. By arbitrarily comparing pairs of parameters, it is possible to determine the relative importance of the 11 parameters. The basic geological data (Table 2) of the 29 DF catchments were applied to calculate the objective weights using the entropy method. Because of the shortcomings of using objective weighting method or subjective weighting method alone, it is necessary to combine the AHP and the entropy method. The combined weights were calculated using Equations (12)–(14).

### 4.4. Hazard Assessment of Debris-Flow

The CM certainty degree of *X_ij_* is calculated based on the forward cloud generator. Meanwhile, the comprehensive certainty degree should be calculated using the following equation:

(18) $$\begin{array}{l} U_{i,p}=\overset{m}{\underset{j=1}{\sum }}\mu_{i,pj}w_{j}\\ i\left(i\;=1,2,\ldots ,n\right);j\left(j=\;1,2,\ldots ,m\right);p\left(p=\;1,2,\ldots ,P\right) \end{array}$$

where *μ_i,pj_* is the *p*-th hazard degree matching the certainty degree of the *j*-th factor of the *i*-th catchment and *w_j_* is the evaluation factor weight of the DF. According to the comprehensive certainty degree *U_p,i_*, it can be determined that the largest comprehensive certainty degree is the hazard degree of the matching DF catchment:

(19) $$k=\;max\{U_{i,}{}_{1},U_{i,}{}_{2},\ldots ,U_{p,n}\}$$

After precise calculation, the certainty values of the 11 parameters of the 29 DF catchments were obtained. The comprehensive certainty was obtained based on Equation (18). The largest comprehensive certainty value matching the hazard degree is the DF catchment hazard degree.

## 5. Results and Discussion

Based on the judgment matrix, *CI* was calculated based on λ_max_ = 11.30, *n* = 11, and *RI* = 1.52; thus, the value of *CR* = 0.0195 was calculated. As the value of *CR* is lower than the threshold (0.1), the consistency of the weighting was affirmed. The AHP weighting results are shown in Table 3. There is only one rainfall monitoring station within the study area, and hence the same rainfall data were used for all the catchments. Furthermore, the location of the study area is remote and few people live here. Because of the characteristics of the entropy method, the weights of the 24-h maximum rainfall and population density were both zero, which means there is no difference between these two parameters in terms of the hazard degree. However, rainfall is one of the most important parameters in DF hazard assessment. This is an example of how the entropy method just reflects the objective results of the investigation and does not reflect expert knowledge. The coefficients are calculated as *α* = 0.695 and *β* = 0.305. The entropy weights and combined weights are shown in Table 3.

An evaluation system for DF hazard assessment, based on a CM that included 11 parameters, has been proposed. The hazard degrees of 29 catchments within the study area and their comparison with the extenics method are listed in Table 4. Among the 29 catchments, the hazard degree of catchment *DD* is high, that of catchments *EG*, *CT*, and *SB* is moderate, and that of the remaining catchments is low. Note that the area of each of the 29 catchments is very small, ranging from 0.013 to 1.803 km^2^. Field surveys showed that no large-scale collapses or landslides existed and that rainfall was not abundant in northern China [50,51]. Thus, the study area was more prone to small-scale and medium-scale DFs [27].

As can be seen in Table 4, the hazard assessment results for the 29 DF catchments, calculated using the proposed approach, are reasonably consistent with the results of the extenics method. The extenics methodwas first proposed by Cai [52] to solve contradictions and incompatibility problems. It is based on matter-element theory and extension mathematics. It concerns not only whether an element belongs to a set but also concerns to what degree it belongs. Thus, it is representative of traditional comprehensive evaluation methods and it has been proven reliable and reasonable for classification [5,53,54,55,56]. In extension theory, *h* (*x*) is the correlation function, which represents the degree an element belongs to a set. If *h* (*x*) ≥ 0, it describes the degree to which the element belongs to the set. If −1 < *h* (*x*) < 0, it indicates that the element does not belong to the set. Nevertheless, the element still has a better chance to be included if the set varies. When *h* (*x*) ≤ −1, it means that the element has no chance to be included by the set. The value describes the degree of the element not belonging to the set [57,58]. The proposed method used for the hazard assessment was considered efficient and believable. The hazard assessment results derived using the two methods correlated very well, except for catchments *EG* and *XFY*, where the CM-based results were moderate and low, respectively. The evaluation accuracy was nearly 93.1%, which indicated that the CM-based approach had precise evaluation capability. The slight deviation was considered acceptable and understandable for a hazard assessment of the actual project.

Compared with the widely used extenics approach, the CM-based approach appeared to offer a more competitive solution for addressing the uncertainties [59]. However, expert knowledge based on field surveys was also a major aspect. As can be seen in Table 4, the results for catchments *EG* and *XFY* derived by the extenics method were low and moderate, respectively. They differed from the analyzed results of the CM-based approach because of inadequate objective data and because expert knowledge is very important in the DF hazard assessment. Many existing methods are vulnerable to the subjective and ambiguous in the process of assessment; however, the CM-based approach could overcome these disadvantages and provide an efficient assessment [60,61]. The determination of the parameter types and their boundaries depends mainly on expert knowledge, but human error does occur, which could affect the accuracy and reliability of the derived results. However, the CM-based approach reduces the deviation because it considers the inherent fuzziness and randomness of such problems. The CM-based approach can directly use the original data without a normalization procedure, effectively avoiding the potential information loss.

In the proposed approach, weights are assigned to the 11 parameters. The subjective weighting method is limited by the extent of expert knowledge, and different researchers have had different experiences and have used different evaluation criteria [45,62,63]. However, the objective weighting method depends on information that does not require expert knowledge. Obtaining sufficient data is laborious and expensive [59], and therefore the combination of the subjective and objective weighting methods was necessary [64]. However, the weights of the 11 parameters calculated based on the combination weighting method may be difficult to be evaluated due to the complexity of the modeling process. Thus, determination of the more accurate weights still needs unremitting efforts.

**Figure 7:**
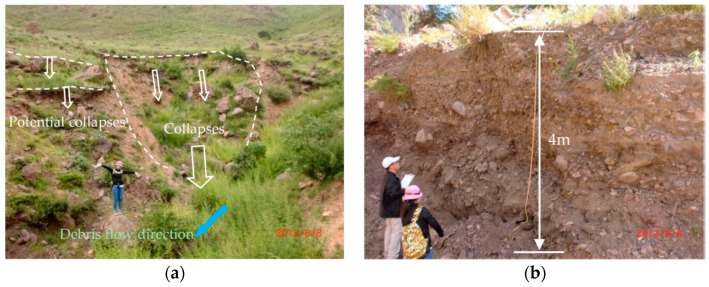
Field survey in *EG* catchment (**a**) collapses and potential collapses on the right side of main channel; (**b**) profile of younger alluvial fan.

The *EG* is assessed as moderate hazardous degree using CM method, while the extenics result is low hazardous. On the right side of main channel in *EG* catchment, there exists a lot of collapses and potential collapses (Figure 7a). The collapsed mud and gravel has been washed along the debris flow in the main channel. Meanwhile, there is existing potential collapses in the neighborhood, which may collapse down induced by the next rainfall. There exists two fans in the *EG* accumulation area. One is an ancient alluvial fan, the other one is a younger alluvial fan. The younger one is shaped as ellipse, whose long axis is 373.5 m and short axis is 90 m. Figure 7b shows the profile of the younger alluvial fan, which is about 4 m thick. The loose material volume of *EG* is 3.276 × 10^4^ m^3^, which is very large among the 29 catchments. The hazardous of *EG* catchment should be moderate instead of low. The assessment result of *XFS* using the extenics method is moderate, while the result of *XFS* using CM is low. In the field, we didn’t find any landslides or collapses in the source area.

**Figure 8:**
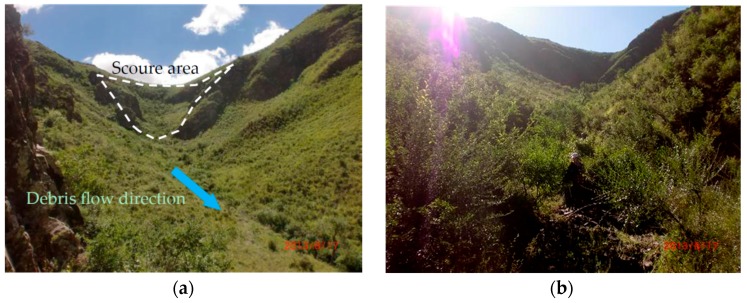
Field survey in *XFS* catchment (**a**) source area and transportation area; (**b**) downstream of the catchment.

**Figure 9:**
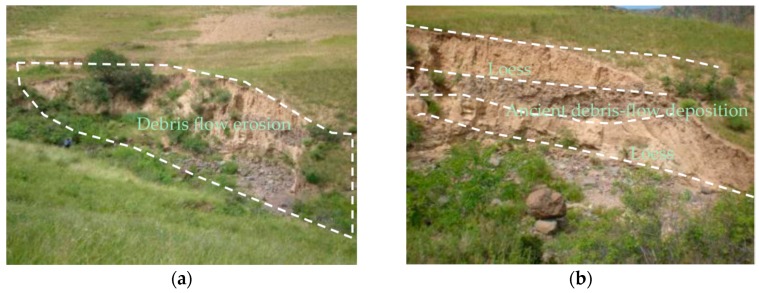
Profiles of ancient debris-flow deposition in *DD* catchment (**a**) panoramic; (**b**) close-up.

Figure 8 shows that the *XFS* catchment is well covered with vegetation. The vegetation cover greatly influence slope behavior at every scale [41]. *XFS* is a small catchment, with less volume of loose material. The hazardous degree of *XFS* catchment should be low. And the proposed CM is more believable. The CM-based hazard assessment result for catchment *DD* was high. Figure 9 shows a cross section that reveals that the left bank of the main channel in catchment *DD* has been subject to processes of erosion and collapse. In the profiles, gravel soil and loess appear alternately, both of which are about 2 m thick. The gravel soil layer is an ancient DF ditch bed. During periods in between DFs, loess was deposited on the surface. Then, a subsequent DF would cut the ancient DF ditch bed. Consequently, it can be inferred that DFs have occurred in the *DD* catchment at least three times. According to Table 2, the outbreak frequency of *DD* catchment is 3.56 times per 100a. Therefore, the three events occurred in 100 years. Therefore, the determination of the hazard assessment of the *DD* catchment as high was reasonable.

In the proposed approach, weights are assigned to the 11 parameters. The subjective weighting method is limited by the extent of expert knowledge, and different researchers have had different experiences and have used different evaluation criteria [45,62,63]. However, the objective weighting method depends on information that does not require expert knowledge. Obtaining sufficient data is laborious and expensive [59], and therefore the combination of the subjective and objective weighting methods was necessary [64]. However, the weights of the 11 parameters calculated based on the combination weighting method may be difficult to be evaluated due to the complexity of the modeling process. Thus, determination of the more accurate weights still needs unremitting efforts.

## 6. Conclusions

Because DFs constitute open, multi-parameter and complex problems, it is difficult to distinguish the randomness and fuzziness of each evaluation parameter. This study used a CM for DF hazard assessment because of the ease with which it can transform the qualitative and quantitative descriptions. According to the 11 selected parameters and their weights, a CM-based approach was established to classify the degree of hazard assessment of DF catchments. Compared with the extenics method, the CM-based hazard assessment approach was validated as credible with an evaluation accuracy of 93.1%. The CM-based approach was shown suitable for a DF hazard assessment, which is a problem of transforming qualitative and quantitative expressions. Furthermore, it considered the uncertainty of randomness and fuzziness in the assessment. The CM-based approach is a type of decision tool that could have wide application to other hazard assessment projects.

This study proposed a new combined weighting method using the distance function for deriving parameter weights, which avoids the disadvantages inherent in using the subjective and objective methods in isolation. The results were proven reasonably consistent with the actual situation.

After a detailed analysis of the original datasets and the proposed approach characteristics, it demonstrates its superiority and validity for the hazard assessment of DF. The results of CM-based approach are reasonable. However, the reliability evaluation result of the cloud model depends on the selection of boundaries of different degrees of an evaluation parameter. These parameters boundaries need expert’s field survey experiences and lots of research to obtain. The comprehensive assessment for DF hazard assessment, how to determine the relevant parameters boundaries more reasonably and accurately should be improved in the future.

The CM-based approach can adequately reflect the hazard degree of DF catchments in the study area Despites the above merits, the CM-based method has its disadvantages. The cloud model requires a lot of input, mainly including expect value (*E_x_*), entropy (*E_n_*), hyper-entropy (*H_e_*) and the number of cloud drops (*N*). The inputs were determined by the classification boundaries of the debris flow hazard assessment, which does not have a verdict. A reasonable determination of the three characteristics of the model should be studied in the future. Since the CM-based approach is still in the phase of theory, it needs to be applied and validated for more study areas in the future.

## Figures and Tables

**Table 1:** Classification of influencing parameters.

Parameters	I	II	III	IV
*x*_1_ (km^2^)	0–0.5	0.5–10	10–35	35–50
*x*_2_ (km)	0–1	1–5	5–10	10–20
*x*_3_ (km)	0–0.2	0.2–0.5	0.5–1.0	1.0–2.0
*x*_4_ (km·km^−2^)	0–5	5–10	10–20	20–40
*x*_5_	0–1.10	1.10–1.25	1.25–1.40	1.40–1.55
*x*_6_	0–0.1	0.1–0.3	0.3–0.6	0.6–1.0
*x*_7_ (mm)	0–25	25–50	50–100	100–300
*x*_8_ (per·km^−2^)	0–50	50–150	150–250	250–350
*x*_9_ (10^4^ m^3^)	0–1	1–10	10–100	100–200
*x*_10_ (%)	0–10	10–50	50–100	100–200
*x*_11_	0–0.1	0.1–0.2	0.2–0.35	0.35–0.85

*x*_1_: catchment area; *x*_2_: main channel length; *x*_3_: maximum elevation difference; *x*_4_: ravine density; *x*_5_: curvature of main channel; *x*_6_: loose material supply length ratio; *x*_7_: twenty-four-hour maximum rainfall; *x*_8_: population density; *x*_9_: loose material volume; *x*_10_: outbreak frequency; *x*_11_: main channel gradient. I: low, II: moderate, III: high, and IV: extremely high.

**Table 2:** Influencing parameter data of 29 debris-flow catchments.

Catchment	*x*_1_	*x*_2_	*x*_3_	*x*_4_	*x*_5_	*x*_6_	*x*_7_	*x*_8_	*x*_9_	*x*_10_	*x*_11_
*DSM*	0.667	1.276	0.445	1.927	1.226	0.55	251.8	0.1	0.608	1.06	0.35
*EG*	1.803	2.119	0.423	3.566	1.072	0.15	251.8	0.1	3.276	0.93	0.26
*XH*	0.034	0.249	0.159	12.44	1.073	0.38	251.8	0.1	0.044	0.97	0.36
*BJZ*	0.143	0.326	0.092	2.28	1.02	0.4	251.8	0.1	0.092	1.06	0.27
*NN5*	0.069	0.281	0.088	6.493	1.156	0.56	251.8	0.1	0.104	0.95	0.32
*LZ*	1.313	2.051	0.391	4.726	1.222	0.6	251.8	0.1	0.77	1.11	0.24
*CT*	1.302	2.051	0.4	4.51	1.145	0.6	251.8	0.1	3.995	2.90	0.27
*DM*	0.5525	0.856	0.098	2.77	1.07	0.3	251.8	0.1	0.7	1.07	0.32
*NNG2*	0.161	0.67	0.139	7.66	1.098	0.2	251.8	0.1	0.193	1.12	0.19
*NNG1*	0.06	0.334	0.057	11.75	1.034	0.2	251.8	0.1	0.055	1.05	0.19
*NNG3*	0.052	0.331	0.061	13.423	1.078	0.08	251.8	0.1	0.058	1.09	0.23
*NNG4*	0.021	0.187	0.057	10.667	1.022	0.08	251.8	0.1	0.0001	1.00	0.34
*L13*	0.099	0.387	0.064	5.21	1.06	0.3	251.8	0.1	0.006	1.00	0.19
*XN*	0.408	0.858	0.215	6.3	1.007	0.4	251.8	0.1	0.393	0.89	0.21
*DD*	1.598	2.591	0.463	3.992	1.328	0.4	251.8	0.1	6.513	3.56	0.19
*XD*	0.211	0.977	0.233	6.93	1.132	0.61	251.8	0.1	1.452	0.64	0.21
*LNCZ*	0.154	0.697	0.129	7.08	1.057	0.2	251.8	0.1	0.217	0.81	0.16
*R4-1*	0.028	0.196	0.097	16.1	1.021	1.0	251.8	0.1	0.0001	1.00	0.14
*R4-2*	0.013	0.26	0.1	20	1.066	1.0	251.8	0.1	0.0001	1.00	0.18
*R5*	0.038	0.226	0.087	11.63	1.092	0.45	251.8	0.1	0.085	0.45	0.27
*LM*	0.069	0.393	0.131	9.975	1.251	0.35	251.8	0.1	0.19	0.76	0.42
*SB*	1.082	1.664	0.355	5.21	1.074	0.4	251.8	0.1	3.38	0.46	0.18
*XFS*	0.064	0.247	0.16	7.78	1.025	0.25	251.8	0.1	0.307	0.62	0.37
*DFS*	0.197	0.633	0.203	7.761	1.347	0.31	251.8	0.1	0.605	0.50	0.18
*XSY*	0.755	1.565	0.258	3.91	1.059	0.53	251.8	0.1	3.244	0.53	0.18
*R11*	0.1142	0.37	0.161	3.616	1.09	0.25	251.8	0.1	0.154	0.83	0.31
*HDH*	0.516	1.25	0.245	4.73	1.136	0.35	251.8	0.1	1.732	0.61	0.14
*R13*	0.078	0.472	0.16	11.22	1.103	0.006	251.8	0.1	0.424	0.83	0.32
*R14*	0.058	0.291	0.155	17.5	1.111	0.35	251.8	0.1	0.146	0.66	0.31

**Table 3:** Weights of influencing parameters in different methods.

Method	*x*_1_	*x*_2_	*x*_3_	*x*_4_	*x*_5_	*x*_6_	*x*_7_	*x*_8_	*x*_9_	*x*_10_	*x*_11_
AHP	0.132	0.053	0.085	0.037	0.017	0.062	0.105	0.017	0.18	0.18	0.132
Entropy	0.287	0.132	0.078	0.069	0.001	0.076	0	0	0.323	0.015	0.019
Combined	0.178	0.076	0.083	0.046	0.012	0.066	0.073	0.012	0.223	0.13	0.098

**Table 4:** Evaluation results of cloud model and comparison.

CCD	k_1_	k_2_	k_3_	k_4_	Cloud Model	Extenics
*DSM*	0.573	0.074	0.067	0.110	Low	Low
*EG*	0.277	0.384	0.079	0.074	Moderate	Low
*XH*	0.624	0.111	0.053	0.080	Low	Low
*BJZ*	0.504	0.152	0.115	0.048	Low	Low
*L5*	0.652	0.156	0.032	0.083	Low	Low
*LZ*	0.478	0.168	0.106	0.093	Low	Low
*CT*	0.306	0.363	0.120	0.093	Moderate	Moderate
*DM*	0.443	0.146	0.057	0.103	Low	Low
*NNG2*	0.346	0.172	0.072	0.072	Low	Low
*NNG1*	0.468	0.116	0.035	0.013	Low	Low
*NNG3*	0.504	0.164	0.011	0.085	Low	Low
*NNG4*	0.382	0.090	0.031	0.017	Low	Low
*L13*	0.425	0.110	0.077	0.082	Low	Low
*XN*	0.463	0.117	0.130	0.073	Low	Low
*DD*	0.153	0.179	0.218	0.089	High	High
*XD*	0.461	0.136	0.042	0.076	Low	Low
*LNCZ*	0.329	0.101	0.036	0.076	Low	Low
*R4-1*	0.442	0.037	0.053	0.015	Low	Low
*R4-2*	0.503	0.102	0.098	0.073	Low	Low
*R5*	0.333	0.086	0.074	0.014	Low	Low
*LM*	0.533	0.099	0.085	0.027	Low	Low
*SB*	0.292	0.396	0.075	0.072	Moderate	Moderate
*XFS*	0.304	0.186	0.096	0.077	Low	Moderate
*DFS*	0.427	0.096	0.113	0.030	Low	Low
*XSY*	0.306	0.113	0.077	0.071	Low	Low
*R11*	0.395	0.104	0.142	0.070	Low	Low
*HDH*	0.452	0.218	0.099	0.063	Low	Low
*R13*	0.425	0.046	0.030	0.041	Low	Low
*R14*	0.393	0.086	0.056	0.020	Low	Low

Note: CCD is the comprehensive certainty degree.
